# Geographical variations and associated factors of defaulting from immunization among children aged 12 to 23 months in Ethiopia: using spatial and multilevel analysis of 2016 Ethiopian Demographic and Health Survey

**DOI:** 10.1186/s12199-021-00984-8

**Published:** 2021-06-12

**Authors:** Mukemil Awol, Zewdie Aderaw Alemu, Nurilign Abebe Moges, Kemal Jemal

**Affiliations:** 1 Department of Midwifery, College of Health Sciences, Salale University, Fitche, Ethiopia; 2 Department of Public Health, College of Health Sciences, Debre Markos University, Debre Markos, Ethiopia; 3 Department of Nursing, College of Health Sciences, Salale University, Fitche, Ethiopia

**Keywords:** Immunization, Defaulters, Children, Spatial, Multilevel, Associated factors, Ethiopia

## Abstract

**Background:**

In Ethiopia, despite the considerable improvement in immunization coverage, the burden of defaulting from immunization among children is still high with marked variation among regions. However, the geographical variation and contextual factors of defaulting from immunization were poorly understood. Hence, this study aimed to identify the spatial pattern and associated factors of defaulting from immunization.

**Methods:**

An in-depth analysis of the 2016 Ethiopian Demographic and Health Survey (EDHS 2016) data was used. A total of 1638 children nested in 552 enumeration areas (EAs) were included in the analysis. Global Moran’s I statistic and Bernoulli purely spatial scan statistics were employed to identify geographical patterns and detect spatial clusters of defaulting immunization, respectively. Multilevel logistic regression models were fitted to identify factors associated with defaulting immunization. A *p* value < 0.05 was used to identify significantly associated factors with defaulting of child immunization.

**Results:**

A spatial heterogeneity of defaulting from immunization was observed (Global Moran’s I = 0.386379, *p* value < 0.001), and four significant SaTScan clusters of areas with high defaulting from immunization were detected. The most likely primary SaTScan cluster was seen in the Somali region, and secondary clusters were detected in (Afar, South Nation Nationality of people (SNNP), Oromiya, Amhara, and Gambella) regions. In the final model of the multilevel analysis, individual and community level factors accounted for 56.4% of the variance in the odds of defaulting immunization. Children from mothers who had no formal education (AOR = 4.23; 95% CI: 117, 15.78), and children living in Afar, Oromiya, Somali, SNNP, Gambella, and Harari regions had higher odds of having defaulted immunization from community level.

**Conclusions:**

A clustered pattern of areas with high default of immunization was observed in Ethiopia. Both the individual and community-level characteristics were statistically significant factors of defaulting immunization. Therefore, the Federal Ethiopian Ministry of Health should prioritize the areas with defaulting of immunization and consider the identified factors for immunization interventions.

## Background

Immunization is the process of inducing an immune response or making our body resistant to an infectious disease, typically by administering a vaccine [1]. Defaulting of immunization refers to missing at least one or more recommended vaccine doses by the national expanded program of immunization (EPI) [2]. Besides the attainment of high coverage of vaccines, receiving complete immunization under 1 year of age is essential to reduce vaccine-preventable diseases among children. It is vital for achieving the Sustainable Development Goals, poverty reduction, and universal health coverage [3].

Globally, 19.4 million children (14%) were not fully vaccinated in 2018, and among them, 13.5 million (70%) did not receive any diphtheria, tetanus, and pertussis (DTP) doses in low-income countries, which is a principal indicator of immunization performance [4]. An estimated 5 million children died each year worldwide [5], nearly 199,000 deaths caused by Haemophilus Influenza type b (Hib), 195,000 by pertussis, 118,000 by measles, 59,000 by neonatal tetanus, 476,000 by pneumococcal disease, and 453,000 by rotavirus [6, 7].

African and Southeast Asian countries are the most affected regions by vaccine-preventable disease, which accounted for 50% of all under-five deaths in 2018 [8, 9]. Ethiopia contributes 46% of the cases, with 51% of deaths from measles among eight eastern African countries [1].

There is a significant disparity in default from immunization between developed and developing countries, ranging from 1% in the Western Pacific to 10% in the African region, the highest (7%) among high-income countries to 3% in low-income countries for the third dose of DTP3 [4].

In Ethiopia, the main barrier of immunization is not the first dose; instead, completion of all recommended doses in the appropriate age. Since the 2016 national report evidenced that, a large number (79.1%) of children age 12 to 23 months took OPV1 (first dose of the oral polio vaccine), from this, only 47.4% of children took MCV1 (last dose of measles vaccine). The overall default rate varied among regions, ranging from 58.6% in the Somali region to 9.3% in Addis Ababa [10].

Similarly, there was marked variation among districts within the same region in Ethiopia. In the South Nation Nationality of People region, Arbaminch town was relatively high (41.2%) [11] compared with the Wonago *district* (20.7%) [12] and Omo *district* (27%) [13]. In the Amhara region, the Mecha *district* contained a high burden (49.1%) [14] compared with the Menjar-Shenkore *district* (18.5%) [15] and Debre Markos town (6.6%) [16].

This geographical variation of defaulting immunization favored the occurrence of vaccine-preventable diseases in clusters or hotspots. For instance, measles' hottest spots are in Africa, and the coldest spots are in the USA and Western Europe [17].

Various studies indicated that the main factors affecting defaulting from immunization were the age of the mother, maternal education, exposure to mass media, antenatal care (ANC), mother’s occupation, birthplace, birth order, place of residence, region, and distance to health care intuitions [18–24].

To minimize defaulting from immunization, Ethiopia has been implementing different strategies such as the combined effect of the Reaching Every District (RED) approach, a health extension program, and implementation of Enhanced Routine Immunization Activities (ERIAs) [25]. A few studies were conducted on child immunization default at national levels in Ethiopia by using EDHS 2011 [26, 27].

Despite this enormous effort to scale up the national vaccination coverage, full immunization remained at 43% in 2019, far from planned coverage of 90% at the national level and 80% in every district level with all vaccines by 2020 [28] with huge variation among regions [29]. Besides, the limitation of those studies was recall bias. In contrast, the 2016 EDHS survey included a health facility recorded data on children’s vaccinations to confirm mother’s report whose children have no vaccination cards to avoid recall bias [30]. Lastly, there were no spatial and multilevel studies to date.

The overall national full immunization coverage after the new vaccines (pneumococcal conjugate vaccine (PCV) and ROTA virus vaccine) has been introduced into the EPI schedule. So, the findings of previous studies are insufficient to capture geographic patterns of defaulting of child immunization. Therefore, to fill this research gap and draw attention to the mostly unexplored contextual factors associated with the defaulting of immunization, we conducted geographical variation and associated factors of defaulting from immunization among children aged 12 to 23 months in Ethiopia.

## Methods

### Study setting

Ethiopia is located in the Horn of Africa and shares a border with Eritrea, Djibouti, Somalia, Sudan, South Sudan, and Kenya. The country covers an area of 1.1 million km^2^ (square kilometer) with geographical diversity, ranging from 4550 meters (m) above sea level down to the Afar depression 110m below sea level, which is comprised of over 80 ethnicities and speaking over 80 different languages [31]. Administratively, Ethiopia is divided into nine regional states, and two city administrations subdivided into 68 zones, 817 districts, and 16,253 kebeles (lowest local administrative units of the country) in the administrative structure of the country [30]. Based on the 2018 world bank report, Ethiopia had a total population of 109 million with a gross national income per capita of US$ 790 [32]. Nearly one seventh (14.9%) of the total population is under the age of 5 years, with an infant mortality rate of 41 per 1000 live births and an under-five mortality rate of 59 per 1000 births [33]. Ethiopia’s health system comprises three tiers: a primary health care unit, a general hospital, and a specialized hospital [34, 35]. Vaccine-preventable diseases are a significant cause of childhood mortality, with under-five mortality of 25,970 deaths due to lower respiratory infections and 14,662 deaths due to diarrheal diseases in 2015 [36].

### Data sources

The data sources were extracted from EDHS 2016, particularly from the under-five children’s file (KR) (http://www.measuredhs.com). The shapefiles for Ethiopia’s administrative boundaries were obtained from the open African website (https://africaopendata.org/dataset/ethiopia-shapefiles).

**Fig. 1:**
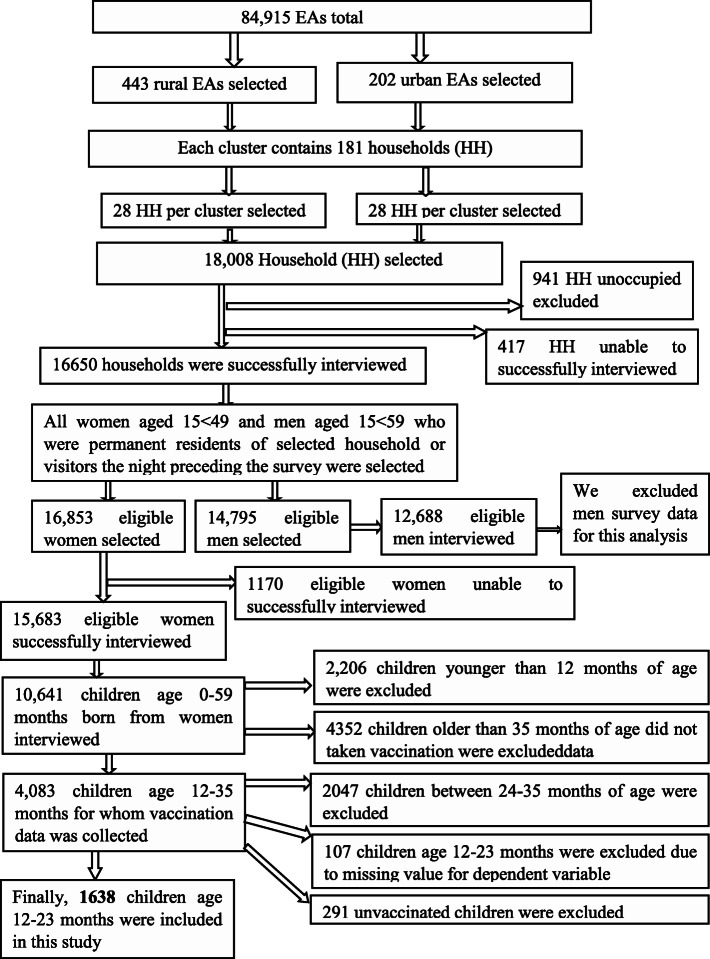
Sampling procedure extracted for children aged from 12 to 23 months from Ethiopian Demographic Health Survey 2016

The EDHS used a two-stage cluster sampling design with rural-urban and regions as strata. A community cluster was defined as a randomly selected area, which contained 150 to 200 households. In EDHS 2016, the actual size of each cluster on average contains 181 households (HH). After that, 28 HH per cluster were selected from 645 clusters drawn by the Ethiopian Central Statistical Agency from its master sampling frame of census 2007 using proportional allocation technique which provided 18,008 HH, and 16,583 reproductive women age 15 to 49 were sampled using a random selection from these clusters [37] (Fig. 1).

Moreover, the EDHS 2016 data set contains only 2036 children aged between 12 and 23 months from 645 sampled clusters that obtained an average of 3 children per cluster. Finally, 1638 children who received at least one dose of the recommended vaccine during their first year of age preceding 2016 with 552 clusters were included for community characteristics. Sixteen children whose geographical locations were not available at the global positioning system (GPS) were excluded from spatial analysis. Additionally, children younger than 12 months, older than 23 months, unvaccinated children, and children who have a missing value for a dependent variable were excluded from this analysis (Fig. 1).

### Study variables

The outcome variable for this study was defaulting from immunization among children aged 12 to 23 months. According to WHO 2017 guideline, children who received at least one dose and missed one or more doses of ten vaccines (one dose of BCG, three doses of pentavalent DPT–HepB–Hib, three doses of PCV, three doses of OPV, two doses of ROTA virus, and one dose of MCV1) at the age of 12 months were considered as defaulter [38].

First, we recoded each variable (recommended vaccinations) as “0” and “1” for children who did not take the recommended doses and those who took, respectively, based on vaccination cards, health facility records, and the mother’s report. Then we added all “0” and “1” and labeled the total as “immunization status”.

The immunization status was recorded as “1” if the child had received all the recommended doses of all vaccinations, categorized as “complete immunization” or “0” if the child had missed one or more doses of vaccinations, and categorized as “default from immunization”.

### Independent variable

Individual-level factors were socioeconomic and demographic characteristics (age of mother, birth order, marital status, child’s sex, number of under-five children, mother’s educational level, partner/husband’s educational level, mother’s occupation, wealth index, exposure to mass media), reproductive health history (antenatal care (ANC) utilization, place of delivery, baby postnatal checkup within 2 months, distance to a health facility, availability of immunization card).

Community-level factors like residence, region, community poverty status, community unemployment, community-women education, community ANC utilization, community distance to a health facility, and community media exposure were the independent variables.

The EDHS did not collect data that can directly describe the clusters’ characteristics except the place of residence and region. Therefore, other common community-level data were generated by aggregating the individual characteristics with our interest in a cluster. The aggregates were computed using the proportion of a given variables’ subcategory in a given cluster since the aggregate value for all generated variables was not normally distributed. It was categorized into two groups (low and high proportion) based on their median values.

The aggregated count data of children whose default immunization were joined to the geographic coordinates based on each EA unique identification code. Location data (latitude and longitude coordinates) were also taken from selected EAs (clusters) in GPS data.

### Definition of terms

*Clustering*: a positive value for Local Moran’s I indicates that a feature has neighboring features with similarly high or low attribute values.

*Outliers*: a negative value for Local Moran’s I indicates that a feature has neighboring features with dissimilar values of an outlier in which a high value is surrounded primarily by low values (HL), and an outlier in which a low value is surrounded primarily by high values (LH). Statistical significance is set at the 95% confidence level.

*Hot spot*: if the z score value is positive, the observed General G index is larger than the expected General G index, indicating high values for the attribute are clustered in the study area.

*Cold spot*: if the z score value is negative, the observed General G index is smaller than the expected index, indicating that low values are clustered in the study area.

*Sampling weights* are adjustment factors applied to each case in tabulations to adjust for differences in the probability of selection and interview between cases in a sample, due to either design or happenstance.

*Log-likelihood ratio*: it assesses the goodness of fit of two competing statistical models based on the ratio of their likelihood.

### Operational definition

*Exposure to mass media*: a frequency of listening to the radio and watching television were considered exposure to mass media in this study by excluding exposure to magazines and newspapers. So, women exposed to either television or radio at least once per week considered exposed, if not exposed at all, taken as not exposed [30].

*Distance to a health facility* was asked to women if the distance was a problem for them to get medical help from health institutions, not limited to child immunization services. There were two categories for this variable “Yes” (the distance was a big problem) or “No” (the distance was not a big problem).

*Antenatal care utilization* was defined as mothers who had at least four antenatal care visits [39].

### Data management and statistical analysis

The EDHS 2016 data were pre-tested before the actual data collection. Data collectors had received training in interviewing techniques, field procedures, the content of the questionnaires, and how to administer both paper and electronic questionnaires; after all, questionnaires were finalized in English, then translated into Amarigna, Tigrigna, and Oromiffa [30]. Since this was secondary data, the data were maintained by processing, editing, and raw coding data, and re-coding, checking its completeness, and cleaning the missing values by running frequencies based on this research’s interest. Sample weights were applied to compensate for the disproportional probability of sampling and non-response rate between the strata that have been geographically defined. A detailed explanation of the weighting procedure can be found in the EDHS final report [30]. Cross tabulations and summary statistics were used to describe the study population. Descriptive and summary statistics were done using STATA version 14 software.

### Multilevel logistic regression

The data for this analysis included 1638 children nested within 552 EAs. Considering this hierarchical nature of the data and the assumption of independence among individuals within the same community and the assumption of equal variance across the community is violated in the nested data. Therefore, flat models could underestimate the effect sizes’ standard errors and lead to bias (loss of power or type I error), affecting the null hypothesis [40].

Multilevel logistic regression models were fitted to identify community and individual-level factors associated with defaulting from immunization.

Four models were developed; Model I (Empty model) was fitted without explanatory variables to test random variability in the intercept and estimate the intraclass correlation coefficient (ICC). Model II examined the effects of individual-level characteristics. Model III examined the impact of community-level variables, and Model IV examined the effects of both individual and community-level characteristics simultaneously. For association (fixed effect) measures, adjusted odds ratio with 95% confidence intervals at *p* value < 0.05 were used to declare statistical significance. For measurements of variation (random effects), ICC, median odds ratio (MOR), and proportional change in variance (PCV) statistics were computed. Model comparison was made based on Akaike information criteria (AIC) and deviance information criteria (DIC). The model with the lowest information criterion was considered to be the best fit model [40].

### Spatial analysis

The aggregated defaulter and completed count data were joined to the geographic coordinates based on each EA unique identification code. Global spatial autocorrelations were assessed with ArcGIS version 10.5 using the Global Moran’s I statistic (Moran’s I) to evaluate whether the pattern expressed is clustered, dispersed, or random across the study areas. Moran’s I values close to − 1 indicated defaulters were dispersed, whereas I values close to + 1 indicated defaulters were clustered, and distributed randomly if I value was zero. A statistically significant Moran’s I (*p* < 0.05) led to the rejection of the null hypothesis and indicated the presence of spatial autocorrelation, and detect the existence of at least one cluster, but not the specific location of the cluster(s) [41].

Hot spot analysis was carried out to identify spatial clusters of defaulting from immunization. Since geographic coordinates were collected at the cluster level, the unit of spatial analyses was DHS clusters. Due to positive global spatial autocorrelation, local spatial association indicators were used to assess clusters and outliers by comparing the values in each specific location with values in neighboring locations. It allows for decomposing the pattern of spatial association into four categories (quadrants) [42]. Finally, we employed Kulldorff’s purely spatial scan statistic method using the Bernoulli probability model in SaTScan version 9.6 software to detect the local spatial clusters of areas with high default of immunization. We declare where spatially significant higher rates of aggregates were founded. Its output presents the hotspot areas in circular windows, indicating the areas inside the windows are higher than expected distributions compared with the areas outside of the cluster windows [43]. We used a maximum 50% of the population at risk for the spatial cluster size. A cluster was statistically significant if a *p* value < 0.05.

For the interpolation, we run the empirical Kriging technique to predict values for areas where data points were not taken.

### Ethical considerations

For this study, ethical clearance was taken from the ethical review committee of Debre Markos University College of Health Sciences (CHS) with approval and a supporting letter. The EDHS 2016 data were then obtained and used with the Central Statistical Agency of Ethiopia’s prior permission. We registered for dataset access and wrote the study’s title and significance on the website after completing a short registration form. Downloading of datasets was done using the accessed website at http://www.measuredhs.com on request with the help of ICF international. Downloading data were used only for this study. The dataset was not passed on to other researchers without the consent of EDHS. All EDHS data were treated as confidential, no need to identify any household or individual respondent interviewed in the survey.

## Results

### Individual-level characteristics of study participants

This study focused on a sample of 1638 unweighted data of children and 1686 weighted children who received at least one specific vaccine during their first year of age before the survey. Three fifth (60.4%) of children had missed one or more doses of recommended vaccine. Two thirds (60.4%) of mothers had no formal education; of them, 40.1% of their children have defaulted from immunization, and nearly half (45.7%) of husbands had no formal education. More than half (53%) of mothers had no jobs, and 33.7% of their children have defaulted from immunization. Nearly one fourth (22.5%) of the household were in the poorest wealth quintile, with 16.8% of children default from immunization.

Regarding media exposure, 80% of women were not exposed to media in a week, and of them, 55.8% of their children have defaulted from immunization. The majority (53%) of the women practice home delivery, with 42.7% of their children default from immunization. Immunization cards were not available for 53.5% of children, and 36% of them were defaulters. In Table 1, the remaining variables were presented.

**Table 1:** Background characteristics of individuals among children aged from 12 to 23 months in EDHS 2016 (unweighted *n* = 1638 and weighted *n* = 1686)

Individual characteristics	N (%) (unweighted)	N (%) (weighted)	Immunization status	
			Defaulted, N (%)	Completed, N (%)
Overall	1638 (100%)	1686 (100%)	1019.164 (60.4%)	666.98 (39.6%)
Age of mother				
15–24 years	460 (28%)	410.50 (24.35%)	239.588 (14.21%)	170.92 (10.14%)
25–34 years	840 (51%)	894.98 (53.08%)	531.346 (31.51%)	363.64 (21.57%)
35–49 years	338 (21%)	380.64 (22.57%)	248.23 (14.72%)	132.42 (7.85%)
Religion				
Orthodox	557 (34.00%)	632.78 (37.53%)	316.07 (18.75%)	316.71 (18.78%)
Muslim	754 (46.03%)	613.27 (36.37%)	424.65 (25.18%)	188.63 (11.19%)
Protestant	284 (17.4%)	367.02 (21.77%)	213.55 (12.66%)	153.48 (9.10%)
Other	42 (2.56%)	73.05 (4.33%)	64.89 (3.85%)	8.16 (0.48%)
Number of under-five children in the household (HH)				
≤ 2	1410 (86.08%)	1467.67 (87.05%)	889.05 (52.73%)	578.65 (34.32%)
> 2	228 (13.92%)	218.44 (12.95%)	130.12 (7.72%)	88.33 (5.24%)
Sex of child				
Male	806 (49.21%)	777.71 (46.12%)	484.9 (28.76%)	292.8 (13.37%)
Female	832 (50.79%)	908.43 (53.88%)	534.26 (31.06%)	374.18 (22.02%)
Birth order number				
First child	311. 24 (19.1%)	322.32(19.12%)	162.94 (9.66%)	159.38 (9.45%)
Second and third	473.3 (28.9%)	526.93(31.25%)	302.03 (17.91%)	224.9 (13.34%)
Fourth and fifth	430.26 (26.26%)	403.33(23.92%)	269.03 (15.96%)	134.3 (7.96%)
Sixth and above	423.2 (25.84%)	433.55(25.71%)	285.15 (16.91%)	148.39 (8.8%)
The child lives with whom				
With respondent	1622 (99.02%)	1,674.29 (99.3%)	1015.84 (60.25%)	658.5 (39.05%)
Elsewhere	16 (%0.98)	11.84 (0.7%)	3.32 (0.2%)	8.51 (0.5%)
Current marital status				
Never union	894 (54.58%)	849.25 (50.37)	9.95 (0.59%)	3.45 (0.21%)
Live to gather	484 (29.55%)	558.21 (33.11%)	960.44 (56.96%)	638.36 (37.86%)
Not living together	260 (15.87%)	278.7 (16.53%)	48.76 (2.89%)	25.15 (1.49%)
ANC utilization				
None-utilized	885 (56.84%)	1008.88 (62.5%)	688.76 (42.67%)	320.12 (19.83%)
Utilized	672 (43.16%)	605.33 (37.5%)	283.62 (17.57%)	321.72 (19.93%)
Postnatal checkup within 6 weeks				
Yes	173 (11%)	154.43 (9.57%)	71.27 (4.41%)	83.17 (5.15%)
No	1385 (89%)	1459.62 (90.43%)	901.11 (55.82%)	558.67 (43.61%)
Distance to the health facility				
Big problem	885 (54.03)	964.649 (57.21%)	638.12 (37.84%)	326.53 (19.37%)
Not a big problem	753 (45.97%)	721.486 (51%)	381.04 (22.6%)	340.44 (20.19%)

*97, observation missing for husband education, *81, observation missing for a postnatal baby check within 2 months, and *81, observation missing for ANC utilization

### Community-level characteristics of study participants

The study included 552 clusters, in which all the children among the age group of 12 to 23 months had lived. In the rest of the 93 clusters, child data for immunization were not taken. Three fourths (76%) of the clusters were in rural with 55.6% default areas, and more than half (57.6%) of clusters were a higher proportion of community poverty status. Of them, 38.4% of children have defaulted from immunization. Nearly half (48.9%) of clusters were a higher proportion of community women unemployed, with 31.2% of children defaulting from vaccination. Three fifths (58.9%) of the clusters was a higher proportion of community of non-educated women, and 39.3% of children have defaulted from immunization. Nearly two thirds (69.5%) of the clusters had a lower proportion of institutional community delivery, with 48% of defaulted children. Additionally, Table 2 illustrated immunization status for the remaining data.

**Table 2:** Background characteristics of communities among children aged from 12 to 23 months in EDHS 2016 (unweighted *n* = 1638 and weighted *n* = 1686)

Community characteristics	N (%) (unweighted)	N (%) (weighted)	Immunization status	
			Defaulted, N (%)	Completed, N (%)
Region				
Tigray	205 (12.52%)	144.7 (8.58%)	50.4 (2.99%)	94.3 (5.59%)
Afar	111 (6.78%)	14.4 (0.85%)	11.9 (0.71%)	2.5 (0.15%)
Amhara	162 (9.89%)	334.03 (19.81%)	188.9 (11.2%)	145.14 (8.61%)
Oromiya	224 (13.68%)	692.16 (41.05%)	478.23 (28.36%)	213.93 (12.69%)
Somalia	178 (10.68%)	60.8 (3.61%)	45.75 (2.71%)	15.09 (0.9%)
Benishangul-Gumuz	136 (8.3%)	17.98 (1.07%)	7.26 (0.43%)	10.73 (0.64%)
South Nation Nationality of People	196 (11.97 %)	352.1 (20.88%)	219.5 (13.02%)	132.58 (7.86%)
Gambela	105 (6.41%)	4.4 (0.26%)	2.5 (0.15%)	1.9 (0.11%)
Harari	113 (6.9%)	4.8 (0.29%)	2.81 (0.17%)	2 (0.12%)
Addis Ababa	100 (6.11%)	51.4 (3.05%)	8.8 (0.52%)	42.62 (2.53%)
Dire-Dawa	108 (6.59%)	9.33 (0.55 %)	3.13 (0.19%)	6.2 (0.37%)
Community-women media exposure				
Lower proportion of media exposure	865 (52.81%)	941.28 (55.83%)	604.23.(35.84%)	337.04.(19.99%)
Higher proportion of media exposure	773 (47.19%)	744.85 (44.17%)	414.9.(24.61%)	329.94.(19.57%)
Community antenatal care utilization				
Lower proportion of utilized	1079 (65.95%)	1234.2 (73.20%)	821.06 (48.7%)	413.13 (24.5%)
Higher proportion of utilized	557 (34.05%)	451.8 (26.80%)	198.04 (11.75%)	253.78 (15.05%)
Community distance to health facility				
Lower proportion of big problem	885 (54.03)	752.77 (44.64%)	380.15 (22.55%)	372.6 (22.1%)
Higher proportion of big problem	753 (45.97%)	933.37 (55.36%)	639.01 (37.9%)	294.35 (17.46%)

### The spatial pattern of defaulting immunization

#### Global spatial autocorrelation

**Fig. 2:**
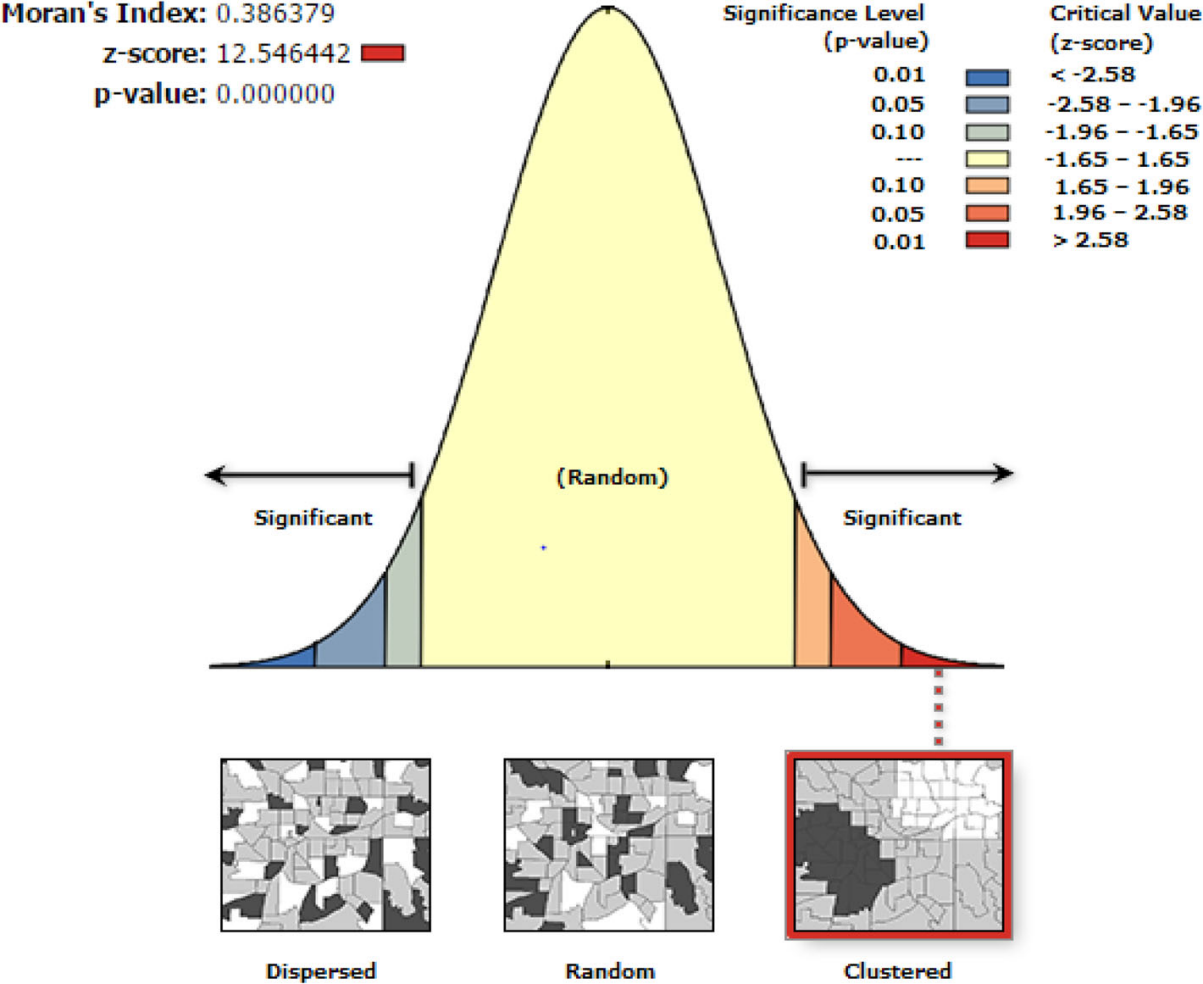
Global spatial autocorrelation based on feature locations and attributes values (immunization defaulters) across the study areas among children aged from 12 to 23 months in EDHS 2016

The global spatial autocorrelation analysis based on feature locations and attribute values revealed a clustering pattern of defaulting immunization across the study areas (Global Moran’s I = 0.386379, *p* value < 0.001) (Fig. 2).

#### Hot spot of defaulting immunization

**Fig. 3:**
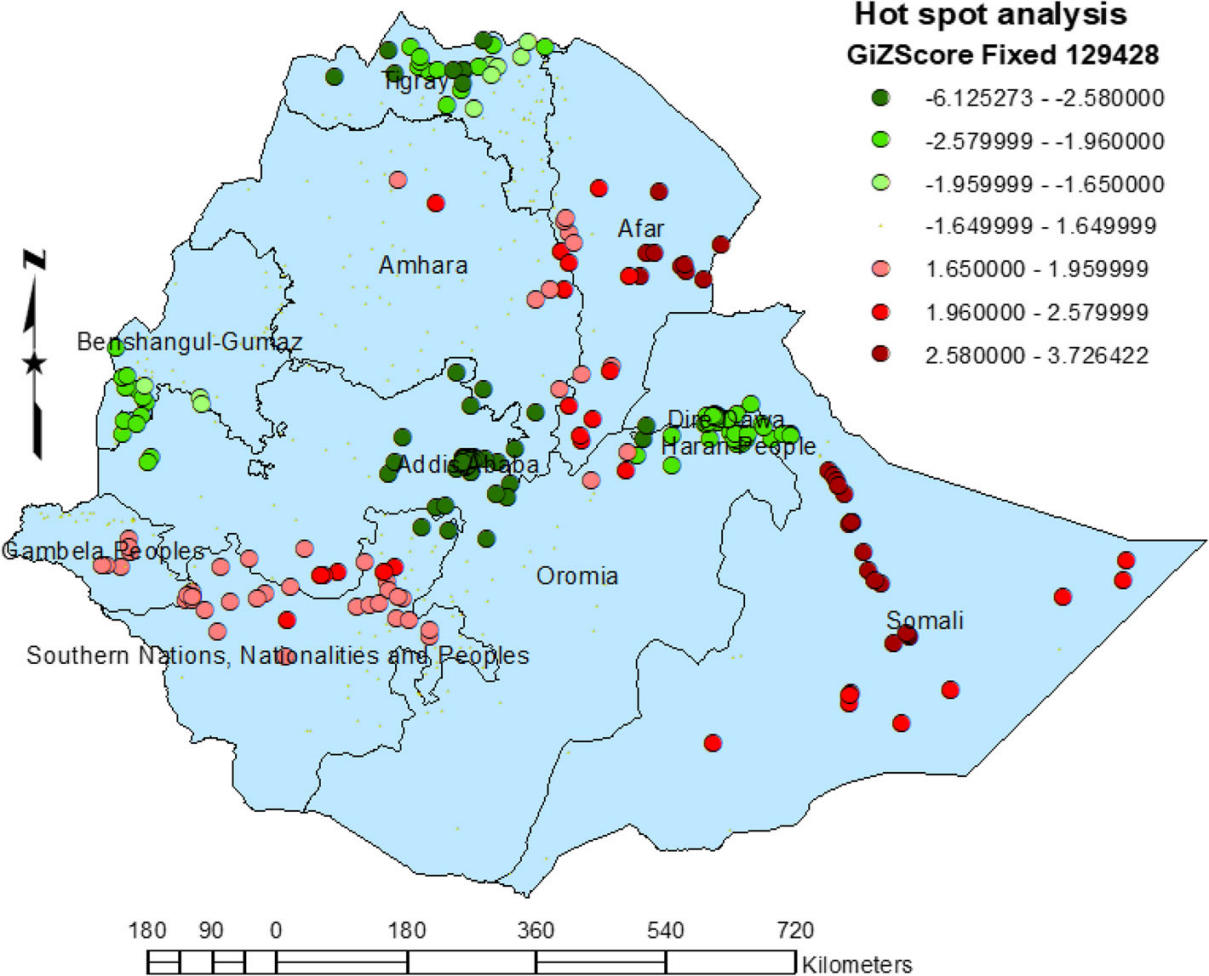
Hot spots of defaulting immunization among children aged from 12 to 23 months in EDHS 2016

There was strong evidence to support spatial clustering in defaulting immunization among children in Ethiopia (Global Moran’s I = 0.386379, z score, *p* value < 0.001). Most of the hot spot areas (high default value) were located in the eastern (Somali) and in northeastern (Afar) parts of the country), followed by some parts of the SNNPR (Konta special woreda), Gambela, Amhara, and Oromiya regions. Conversely, most cold spot areas (low default rates) were located in Addis Ababa, Tigray, Dire Dawa, Harari, and Benshangul-Gomez (Fig. 3).

### Cluster and outlier analysis (Anselin’s Local Moran’s I)

**Fig. 4:**
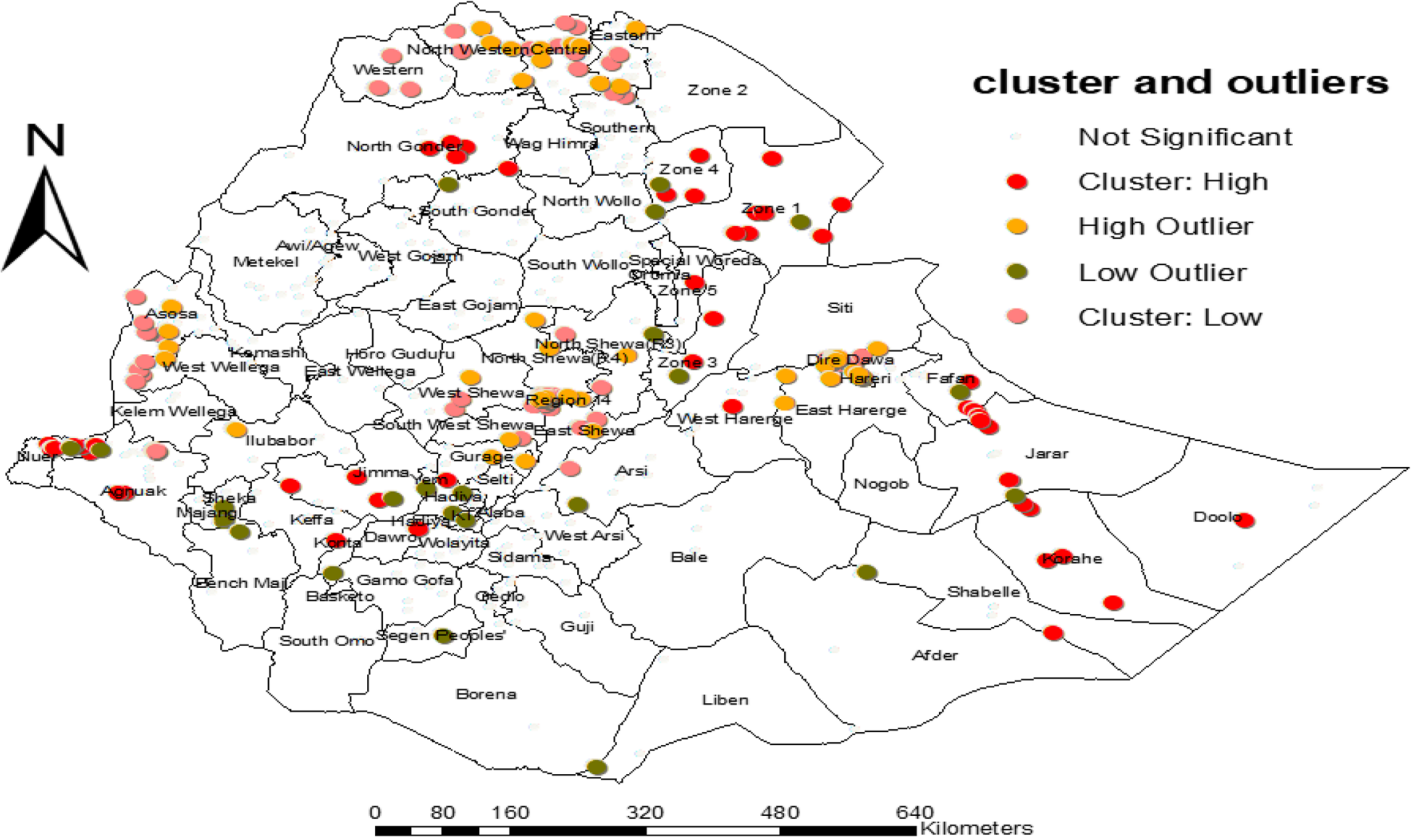
Clusters of defaulting immunization among children aged from 12 to 23 months in EDHS 2016

The spatial cluster of autocorrelation on defaulting immunization indicates that statically negative autocorrelation (with high cluster, low outlier) was observed in the Afar region (zone 3, zone 4), Amhara region (South Gonder), Somalia (Fafan, Korahe, Jarar), Oromiya (Jimma), SNNPR, and Gambela (Nuer). Statistically negative autocorrelation (with high outlier, low cluster) was observed in the Tigray region (northwestern, central, and eastern), Dire Dawa, Harari, Addis Ababa (zone 4), Benishangul-Gumuz, and Oromiya (West Wallega, North Shewa, East Shewa, and West Shewa), while positive autocorrelation with high cluster and high outlier was observed in Oromiya and Amhara regions as well as that with low outlier and low cluster was observed in Gambela (Agnuak) (Fig. 4).

### Interpolation result

**Fig. 5:**
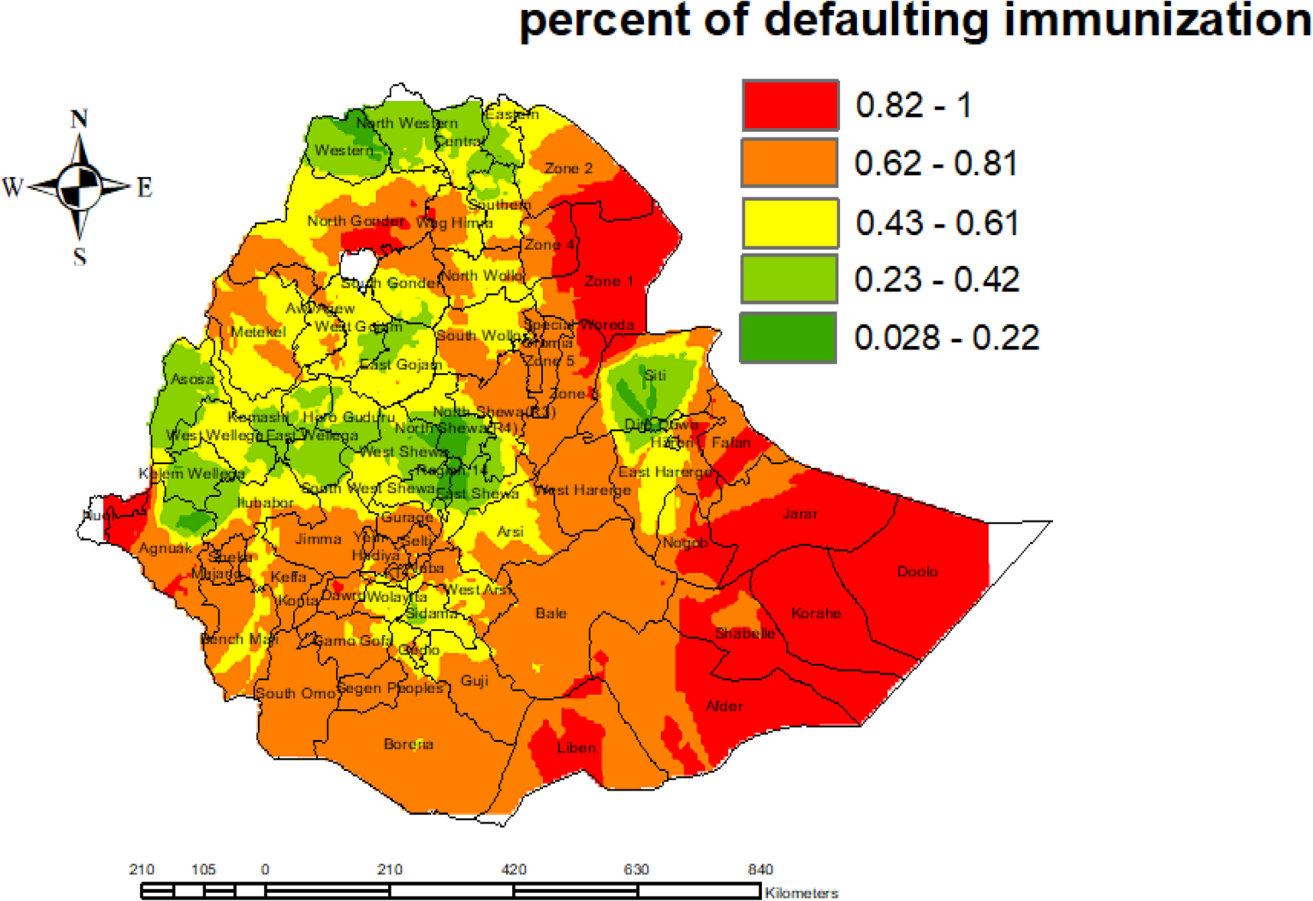
Interpolation of defaulting from immunization among children aged from 12 to 23 months in EDHS 2016

We estimated the spatial autocorrelation from defaulting immunization for areas where data points were not taken across Ethiopia by using the kriging method. More than 82% of children who live in an area with red color have defaulted from immunization, whereas less than 22% of children who resided in an area with green color have defaulted from immunization (Fig. 5).

### SaTScan spatial analysis

**Fig. 6:**
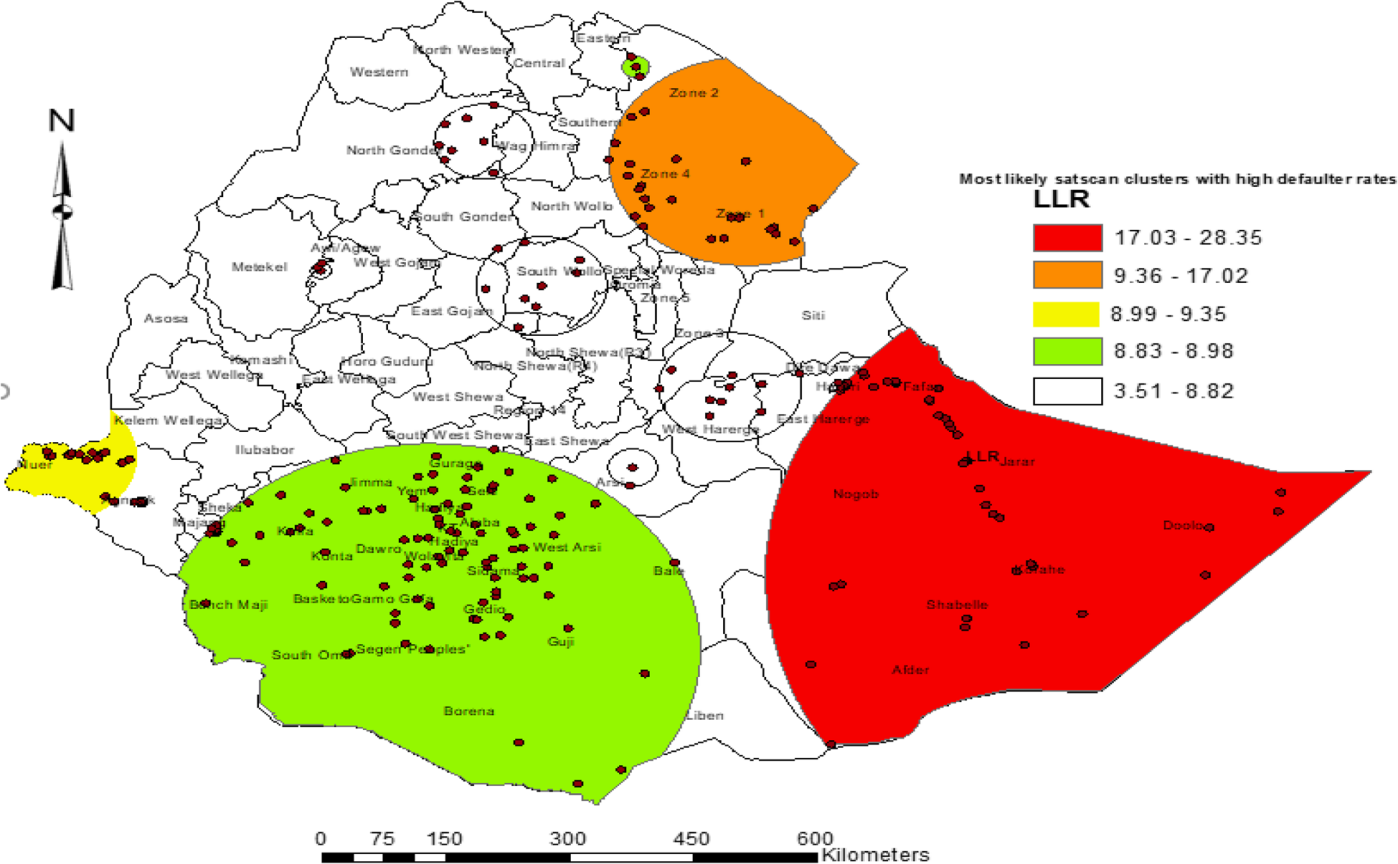
Spatial clustering of areas with defaulting from immunization among children aged from 12 to 23 months in EDHS 2016

The SaTScan spatial analysis detected four groups of statistically significant SaTScan cluster areas with high immunization defaulters. This means that the prevalence of immunization default was higher inside the SaTScan circular window compared with that outside the SaTScan window. The most likely primary SaTScan cluster of high default areas was detected (LLR = 28.35, *p* < 0.001 in Eastern Ethiopia, specifically in Afder, Shabelle, Korah, Doolo, Nogob, Jarar, and Fatan administrative zones of the Somali region. The first secondary most likely spatial SaTScan cluster was in the Afar region (LLR = 17.03, *p* < 0.001), specifically in Zone 1, Zone 2, and Zone 4 administrative zones (Fig. 6).

The primary cluster centered at 6.559519 N, 46.154797 E with 531.31-km radius, RR of 1.58, and LLR of 28.35 at *p* < .001 showed that children within the area had a 58% higher risk of defaulting from immunization than children outside the area. The first secondary clusters were centered at 12.53287 N, 41.1649 E with 165.63-km radius, RR of 1.63, and LLR of 17.02, *p* < 0.001 showed that children within the area had a 63% higher risk of defaulting from immunization than children outside the area (Table 3).

**Table 3:** The most likely clusters from a purely spatial scan statistic (Bernoulli model) of defaulting immunization among children aged from 12 to 23 months in EDHS 2016

Clusters	Regional location (zones)	No. of clusters	No. of population	No. of cases	Coordinates	Radius (km)	Relative risk	LLR	*P* value
Primary cluster	Somali region (Afder, Shabelle, Korah, Doolo, Nogob, Jarar, and Fatan) and Harari people	44	147	123	(6.559519 N, 46.154797 E)	531.31	1.58	28.35	0.001
1st secondary cluster	Afar region (Zone 1, Zone 2, & Zone 4)	24	63	56	(12.532879 N, 41.164991 E)	165.63	1.63	17.02	0.001
2nd secondary cluster	Gambella region (Nuer & Agnuak)	14	43	37	(8.268721 N, 33.486779 E)	104.98	1.57	9.35	0.034
3rd secondary cluster	SNNPR and Oromiya (Jimma, East Shewa, west Arsi, Goji people, and a part of Bale)	99	312	207	(5.546952 N, 37.666334 E)	327.57	1.25	8.98	0.044

### Multilevel logistic regression analysis

As shown in the results of multilevel logistic regression analysis in Table 4, the empty model (Model I) revealed that there was a considerable variation in the odds of defaulting immunization between communities (ICC = 34%), which implies 34% of the total variance in the defaulting immunization was attributed to differences between communities.

**Table 4:** Multilevel binary logistic regression analysis of individual- and community-level factors among children aged from 12 to 23 months in EDHS 2016

Individual and community characteristics	Null model	Model II AOR (95%CI)	Model III AOR (95% CI)	Model IV AOR (95% CI)
Respondent age				
15–24 years	-	1	-	1
25–34 years	-	0.98 (0.55, 1.74)	-	0.95 (0.53, 1.70)
35–49 years	-	1.17 (0.54, 2.57)	-	1.33 (0.59, 2.97)
Birth order				
First birth	-	1	-	1
Second and third	-	0.95 (0.50, 1.84 )	-	0.86 (0.45, 1.63)
Fourth and fifth	-	1.19 (0.59, 2.42)	-	1.05 (0.52, 2.11)
Six and above	-	1.01 (0.41, 2.51)	-	0.82 (0.33, 2.01)
Antenatal care utilization				
< 4 visit	-	1.63 (1.02, 2.59 ) *	-	1.26 (0.74, 2.14)
> 4 visit	-	1	-	1
Women’s occupation				
No job	-	1.21 (0.80, 1.81)	-	1.23 (0.77, 1.97)
Has job	-	1	-	1
Distance to the health facility				
Big problem	-	1.23 (0.83, 1.84)	-	0.86 (0.53, 1.40)
Not a big problem	-	1	-	1
Partner /husband’s educational level				
No education	-	0.52 (0.16, 1.64)	-	0.48 (0.15, 1.50)
Primary	-	0.50 (0.16, 1.50)	-	0.44 (0.15, 1.32)
Secondary	-	0.45 (0.14, 1.47)	-	0.47 (0.14, 1.60)
Higher	-	1	-	1
Do not know	-	5.53 (0.74, 41.06)	-	7.56 (0.10, 57.72)
Wealth index				
Poorest	-	2.38 (0.86, 6.58)	-	1.79 (0.50, 6.37)
Poorer	-	1.81 (0.75, 4.33)	-	1.41 (0.45, 4.46)
Middle	-	2.21 (0.93, 5.23 )	-	182 (0.61, 5.42)
Richer	-	1.45 (0.63, 3.37)	-	1.32 (0.46, 3.79)
Richest	-	1	-	1
Women’s educational level				
No education	-	4.16 (1.09, 15.89)*	-	4.23 (1.17, 15.78)*
Primary	-	2.94 (0.79, 10.87)	-	3.06 (0.87, 10.73)
Secondary	-	1.52 (0.40, 5.71)	-	1.64 (0.45, 5.97)
Higher	-	1	-	1
Media exposure				
Not exposed at all	-	1.51 (0.49, 4.71)	-	1.81 (0.54, 6.10)
Exposed to either radio/TV	-	1.17 (0.39, 3.53)	-	1.33 (0.40, 4.43)
Exposed to both radio and TV	-	1	-	1
Religion				
Orthodox	-	1		1
Muslim	-	1.39 (0.72, 2.71)	-	1.28 (0.65, 2.54)
Protestant	-	0.82 (0.40, 1.60)	-	0.86 (0.41, 1.81)
Other	-	4.87 (1.18, 20.10)	-	2.10 (0.25, 20.74)
**Place of delivery**				
Home	-	1.60 (1.04, 2.46)*	-	1.33 (0.82, 2.16)
Health facility	-	1	-	1
Other	-	0.39 (0.10, 1.48)	-	0.35 (0.08, 1.52)
Region				
Tigray	-	-	1	1
Afar	-	-	8.39 (2.39, 29.81)**	7.53(2.06, 27.45)**
Amhara	-	-	2.38 (1.12, 5.03)*	2.13(0.98, 4.63)
Oromiya	-	-	3.07 (1.31, 7.12)*	3.70 (1.52, 9.03)*
Somalia	-	-	4.82 (1.59, 14.64)**	4.39 (1.31, 14.69)*
Benishangul	-	-	0.83 (0.34, 2.00)	0.79(0.31, 1.99)
SNNPR	-	-	3.10 (1.33, 7.24) **	3.44 (1.37, 8.67)*
Gambela	-	-	3.31 (1.25, 8.74)*	3.151 (1.11, 8.92)*
Harari	-	-	2.94 (1.10, 7.93)*	2.94 (1.02, 8.48)*
Addis Ababa	-	-	0.87 (0.30, 2.53)	1.44 (0.42, 4.91)
Dire Dawa	-	-	0.8 (0.27, 2.43)	1.08 (0.33, 3.56)
Residence				
Rural	-	-	1.15 (0.46, 2.86)	0.75 (0.23, 2.41)
Urban	-	-	1	1
Community media exposure				
Lower proportion of exposed	-	-	1.20 (0.70, 2.05)	0.96 (0.53, 1.75)
Higher proportion of exposed	-	-	1	1
Community distance to health facility				
Lower proportion of big problem	-	-	1	1
Higher proportion of big problem	-	-	1.55 (0.96, 2.51)	1.60 (0.92, 2.78)
Community poverty status				
Lower proportion of poverty	-	-	1	1
Higher proportion of poverty	-	-	1.32 (0.76, 2.28)	1.29 (0.70, 2.38)
Community-women educational level				
Lower proportion of no education	-	-	1	1
Higher proportion of no education	-	-	1.22 (0.76, 1.96)	1.05 (0.61, 1.81)
Community antenatal care (ANC) utilization				
Low proportion of ANC utilization	-	-	1.37 (0.82, 2.29)	1.16 (0.67, 2.02)
Higher proportion of ANC utilization	-	-	1	1
Community unemployment rate				
Lower unemployment rate	-	-	1	1
Higher unemployment rate	-	-	0.79 (0.49, 1.26)	0.69 (0.39, 1.21)

Notes: * *p* < 0.05; ** *p* < 0.01, 1 = Reference category

In Model II, only individual-level variables were added. The results showed that women’s educational level, antenatal care utilization, and delivery place were significantly associated with defaulting of immunization (Table 4).

In Model III, only community-level variables were added to assess how much the variation in defaulting of immunization is explained by community variation. The result revealed that children residing in Afar, Somalia, SNNPR, Oromiya, Amhara, Harari, Gambela, and place of delivery were significantly associated with defaulting of immunization (Table 4).

The final model (Model IV) included both the individual and community-level characteristics simultaneously depicted that women’s educational level and region were significantly associated with defaulting immunization (Table 4).

### Interpretation of Model IV (mixed model result)

Children from mothers who had no formal education have 4.23 times (AOR = 4.23; 95% CI: 1.17, 15.78) higher likelihood of defaulting for immunization than children whose mothers had higher education. Those children who live in the Afar region have 7.53 times (AOR = 7.53; 95% CI: 2.06, 27.45), in the Oromiya region 3 times (AOR 3.070; 95% CI: 1.52, 9.03), in the Somali region 4.4 times (AOR = 4.39; 95% CI: 1.31, 14.69) more likely to default from immunization as compared with children who were residing in the Tigray region. Additionally, children who live in SNNPR (AOR = 3.44; 95% CI: 1.37, 8.67), of the Gambella people (AOR = 3.32; 95% CI: 1.11, 8.92) and in the Harari region (AOR = 2.94; 95% CI: 1.02, 8.48) had more likely to default immunization as compared with children who were residing in the Tigray region (Table 4).

### Measures of variation (random-effects) and model fit statistics

As the multilevel logistic regression analysis results described in Table 5, Model I revealed statistically significant variation in defaulting of immunization across communities. One third (34%) of the variation in the odds of defaulting immunization is attributed to community-level factors (ICC = 34%).

**Table 5:** Measures of variation (random intercept models) and model fit statistics in defaulting immunization among children aged from 12 to 23 months in EDHS 2016

	Null model	Model II	Model III	Model IV
Random effect result				
ICC (%)	34%	24.4%	20.8%	18.7%
PCV (%)	Reference	37.3%	49%	55.6%
MOR	3.5	2.7	2.4	2.3
Model fit statistics				
Log-likelihood	− 1078.19	− 924.53861	− 1007.379	− 893.4072
AIC	2160.381	1895.077	2060.757	1872.814
DIC	2156.28	1849.0782	200.758	1786.8144

Note an increased risk (in median) that one would have if moving to a neighborhood/cluster with a higher risk

After adjusting the model for individual-level factors (Model II), about 37.3% of the variation in the odds of defaulting immunization was attributed to the individual level factors (PCV = 37.3%), and 24.4% of the variance in defaulting of immunization was attributed to community-level factors (ICC = 24.4%) (Table 5).

Model III, which was adjusted for community-level factors, revealed that the community-level factors explained 49% of the variability in the odds of defaulting of immunization (PCV = 49%), and 21% of the variation among the clusters was attributed to community-level factors (ICC = 21%) (Table 5).

The final best-fit model (model IV) was adjusted for both individual and community-level factors simultaneously. In this final model, about 18.5% of the variability among communities in the odds of defaulting immunization was due to the community-level factors (ICC = 18.7%) and 55.6% of the variance in the odds of defaulting immunization (PCV = 55.6%) across communities was attributed to both individual and community-level factors (Table 5).

Including both individual- and community-level factors reduced the unexplained heterogeneity in defaulting immunization between communities from MOR of 3.5 in the null model to the MOR of 2.3 in the final model, which equals 1.2. This showed that the likelihood of having a default for immunization increased by 20% when children moved from low- to high-risk neighborhoods (Table 5).

### Model fit statistics

As shown in Table 5 below, a small number of AIC and DIC and a large number of LLR are in Model IV, indicating that the explanatory value of the model increases for Model IV. In other words, Model IV explained the determinants better than Models II and III; this makes the final model the best-fitted model than others (Table 5).

## Discussion

The current study found that two thirds (60.5% (95% CI = 0.58, 0.62) of Ethiopian children among the age group of 12 to 23 months were missed one or more doses of recommended vaccine. The proportion of defaulter in this study is comparatively higher than in previous studies in the Democratic Republic of the Congo [20], in Togo [44], and Malesia [45]. This finding is also higher when compared with the studies done in Ethiopia, Arbaminch [11], in West Showa Zone [46], and Mecha *district* [14]. The variation might be due to the use of national data for the current study while the previous studies were district or used a small sample size. In addition to the sample size, also a discrepancy of immunization coverage from region to region in Ethiopia and socioeconomic and sociocultural perspectives may have affected the community immunization coverage [47]. On the other hand, this finding is lower than the previous studies done in Ethiopia [26, 27, 48], and Nigeria [21]. This progressive improvement might be due to expanding basic health care service with the combined effect of an organized health extension program and implementation of Enhanced Routine Immunization Activities [25].

In this study, the distribution of defaulting from immunization among children 12 to 23 months of age was varied in the country. The same coverage of defaulters was aggregated in specific areas. SaTScan spatial analysis detected four groups (181 clusters) of statistically significant, most likely SaTScan clusters of high default coverage areas.

The high burden and high risk of children defaulted from immunization were detected mainly in the Somalia region, followed by Afar, Gambella, and SNNPR. This may be due to differences in health service accessibility and utilization and sociocultural differences in the community. Studies identified that Somalia, Afar, Gambella, and SNNPR regions have sociodemographic variation, low healthcare-seeking behavior, low immunization coverage, and low vaccine uptake due to lack of permanent residence as the community is nomadic and pastoralist inhabitants [48, 49]. In remote regions, the lack of strengthening health care systems is contributed to defaulting from immunization because most of the vaccine and supplies are stockouts, poorly arranged immunization services or appointments, lack of tracking systems to obtain defaulters, and poor maternal counseling [47].

Furthermore, it might be due to discrepancies in the birth interval and fertility rate across regions. The median birth interval ranges from 25.1 months in Somali to 47.6 months in Addis Ababa, while a fertility rate of 5.7 per woman in the Somalia region and 5.5 in the Afar region, which is higher compared with 1.8 per woman in the Addis Ababa region. Emphasis on birth spacing can have an impact on vaccination demand and vaccination-seeking behavior among parents [50, 51]. Children with low parity households in the UK were more likely to be vaccinated than those in high-parity families [52].

Maternal non-education was identified as a factor for defaulting from immunization in the current study. This finding is supported by previous studies done in Ethiopia [23, 26], Kenya [53], sub-Saharan Africa [54], the Democratic Republic of the Congo [20], and Togo [44]. This may be because education may provide greater knowledge of health care utilization and the ability to respond to new knowledge more rapidly. Educated mothers might be more aware of the importance of immunization and may choose health care services that generate better health status for their children.

From the community level characteristics, the geographical region was a significant factor in defaulting immunization. Children living in Afar, Somali, SNNPR, Oromiya, Gambella, and Harari regions were more likely to default immunization than children in Tigray. This finding is consistent with previous studies done in Ethiopia that found default from immunization is common in Afar, Amhara, Oromiya, Somalia, SNNPR, Gambella, and Harari regions [27, 48]. This might be the inconsistency of receiving continuum care among all regions. For instance, institutional delivery was lowest in Afar (15%) followed by Somali (18%) whereas high in Addis Ababa (97%) [10]. The proportion of women who received postnatal check-ups on the 2nd day after delivery widely varies by region, from a low of 12.6% in Somalia and 13.4% in Afar to a high of 55% in Addis Ababa [10]. Mothers, who can receive better continuity of care through a health facility during postnatal health services were more likely to complete vaccination [55]. Again, regional variation could be linked with differences in vaccine supply, availability of health care providers, and accessibility of health facilities [48, 49]. Hence, these regional divergences tend to affect the range of child immunization across the country.

In contrast to the previous studies, factors like distance to the health facility, residence, community ANC follow-up, and media exposure were not significantly associated with this study. This might be due to effective strategies of the country that combines the effect of the Reaching Every District (RED) approach, a health extension program, and implementation of Enhanced Routine Immunization Activities (ERIAs) [25]. The health extension workers are currently providing a package of integrated essential health services, which immunization services are a major component. Additionally, the application of new comprehensive strategies to scale up immunization coverage and outreach services for communities living away from 5 km to the static health facilities with mobile immunization teams were continuously implemented [26, 27]. These effective initiatives may enhance the community utilization of health care seeking and ANC follow-up, and exposure to media was improved; full immunization coverage was increased from 24.3% in 2011 to 38.3% in 2016 EDHS [48, 49]. Even though immunization coverage increased in Ethiopia through time, the defaulter from immunization was highly varied among administrative regions, ranging from the highest defaulter in Afar (91.2%) to the lowest defaulter found in Addis Ababa (13.2%) [49].

### Limitation

This study cannot determine causality because of the cross-sectional study design. The other limitation of this study was that some random effect values not explained by the predictors including beliefs, cultural tradition, knowledge, attitude, and practices (KAP) were found to be barriers to child immunization from a previous study conducted in Southern Ethiopia in 2013. Due to the irregular shape of the earth, a circular SatScan window may miss intervention areas. There is no data in 2016 EDHS, that measure the distance of the household to the health facility. It was asked whether the distance to the health facility was a big problem or not. Lastly, the sample size may not be adequate to generalize at the cluster level.

### Strength

Regarding the strength of the methodology to consider the hierarchical nature of the EDHS data, a two-level mixed-effects logistic regression was used to handle both the fixed effects of individual and community factors and random effects to explain the between-cluster variations simultaneously. Furthermore, it is noted that EDHS data often collects individual data; it does not collect data that describe the clusters directly except region and place of residence. As a result, this study endeavored to generate variables that can characterize communities by aggregating individual data into cluster values. This enabled the study to test whether community-level factors could influence the defaulting of child immunization, in addition to individual-level factors. The other strength of this study is with estimation adjustments for representativeness of EDHS data, such as applying weighting of data during the description of background characteristics of the study population and considering sample designs during the analysis of cross tabulation, results obtained based on EDHS data are assumed to be representative of the Ethiopian population.

## Conclusion

This study observed a clustered pattern of areas with high default immunization coverage in Ethiopia. Statistically significant local clusters of high default areas were detected in the country’s Somali, Afar, Gambella, and Oromiya regions. The individual-level characteristics (women’s educational level) and community-level characteristics (geographic region) were statistically significant factors of defaulting immunization. Therefore, it is good if the federal ministry of health and other concerned child health programmers give priority to the areas with high default coverage identified in this study. It is also better to consider the individual- and community-level determinant factors that may help planners, policy, and decision makers emphasize individuals and communities. Finally, Ethiopian Central Statistical Agency is better to emphasize maximizing the number of children with vaccination status in its cluster to increase representativeness.

## Data Availability

The datasets generated and/or analyzed during the current study are available in the Measure EDHS Program repository (https://www.dhsprogram.com/data/dataset_admin) to all registered users.

## Abbreviations

BCG: Bacillus Calmette–Guerin
DHS: Demographic and Health Survey
DPT–HepB–Hib: Pentavalent vaccine (for diphtheria, pertussis, tetanus, hepatitis B, H. influenza type b)
EA: Enumeration area
EDHS: Ethiopian Demographic and Health Survey
EPI: Expanded Program of Immunization
GIS: Geographic Information Systems
ICC: Intraclass correlation
LISA: Local indicator of spatial association
MOR: Median odds ratio
PCV: Proportional change in variance
USAID: United States Agency for International Development
